# Adrenal Insufficiency and Glucocorticoid Use During the COVID-19 Pandemic

**DOI:** 10.6061/clinics/2020/e2022

**Published:** 2020-06-08

**Authors:** Madson Q. Almeida, Berenice B. Mendonca

**Affiliations:** I Unidade de Suprarrenal & Unidade de Endocrinologia do Desenvolvimento, Laboratorio de Hormonios e Genetica Molecular (LIM/42), Servico de Endocrinologia e Metabologia, Hospital das Clinicas HCFMUSP, Faculdade de Medicina, Universidade de Sao Paulo, Sao Paulo, SP, BR; II Servico de Endocrinologia, Instituto do Cancer do Estado de Sao Paulo (ICESP), Faculdade de Medicina FMUSP, Universidade de Sao Paulo, Sao Paulo, SP, BR

**Keywords:** Adrenal Insufficiency, Glucocorticoid, SARS-CoV-2, COVID-19

## Abstract

The coronavirus disease 2019 (COVID-19) is an emerging pandemic challenge. Acute respiratory distress syndrome (ARDS) in COVID-19 is characterized by a severe cytokine storm. Patients undergoing glucocorticoid (GC) replacement therapy for adrenal insufficiency (AI) represent a highly vulnerable group that could develop severe complications due to the SARS-CoV-2 infection. In this review, we highlight the strategies to avoid an adrenal crisis in patients with AI and COVID-19. Adrenal crisis is a medical emergency and an important cause of death. Once patients with AI present symptoms of COVID-19, the dose of GC replacement therapy should be immediately doubled. In the presence of any emergency warning signs or inability to administer oral GC doses, we recommend that patients should immediately seek Emergency services to evaluate COVID-19 symptoms and receive 100 mg hydrocortisone by intravenous injection, followed by 50 mg hydrocortisone intravenously every 6 h or 200 mg/day by continuous intravenous infusion.

## INTRODUCTION

The coronavirus disease 2019 (COVID-19), caused by the severe acute respiratory syndrome coronavirus 2 (SARS-CoV-2), is an emerging pandemic (1). In severe cases, patients with COVID-19 develop a type of acute respiratory distress syndrome (ARDS) approximately 8-9 days after symptom onset (2). The SARS-CoV-2 infection triggers a local immune response in lung cells, recruiting macrophages and monocytes. SARS-CoV-2 induced-ARDS is characterized by a severe cytokine storm. The overproduction of early response proinflammatory cytokines (tumor necrosis factor [TNF], interleukin [IL]-6, and IL-1β) leads to vascular hyperpermeability and multiorgan failure (3).

Besides inflammation, patients with COVID-19 are susceptible to the development of microthrombosis and disseminated intravascular coagulation (4,5). Acute pulmonary embolus has been reported in around 20% of severe COVID-19 infections (6). Additionally, cerebral infarcts in multiple vascular territories, as well as ischemia in the lower and upper limbs, have been documented in patients with COVID-19 (7). The initial coagulopathy of COVID-19 reveals a prominent elevation of D-dimer and fibrin/fibrinogen degradation products. Coagulation is activated by the inflammatory response through several procoagulant pathways, resulting in vascular endothelial injury (8).

In the face of the COVID-19 pandemic, patients undergoing glucocorticoid (GC) therapy represent a highly vulnerable group that could develop severe complications due to the SARS-CoV-2 infection. We can classify patients receiving GC therapy into two groups: 1) Patients with primary, secondary and tertiary adrenal insufficiency (AI) receiving GC replacement therapy; and 2) Patients receiving supraphysiological GC doses due to its potent anti-inflammatory and immunosuppressive effects.

Cortisol is a glucocorticoid hormone synthesized and secreted by the zona fasciculata of the adrenal gland cortex. Under physiological situations, cortisol (called “stress hormone”) has the main function of preparing our body for a “fight or threat” reaction. Cortisol modulates the stress response, blood glucose levels, blood pressure, immune system, and inflammatory processes. Therefore, cortisol deficiency leads to an altered innate immune cell response to infection, increased production of inflammatory cytokines, loss of the cortisol synergic action to maintain the vasopressor effects of catecholamines, and decreased metabolic energy (reduced gluconeogenesis and circulating free fatty acids and amino acids) (9,10).

### Adrenal insufficiency

AI is attributed to a failure of cortisol production by the zona fasciculata of the adrenal cortex (primary AI or Addison disease) or by a deficiency of adrenocorticotropin hormone (ACTH, secondary AI) and/or corticotropin-releasing hormone (CRH, tertiary AI). Primary AI is mainly caused by autoimmune adrenalitis or infectious diseases (tuberculosis, paracoccidioidomycosis, HIV) (10,11). Hemorrhage or thrombosis are rare causes of primary AI, including thrombocytopenia, Waterhouse-Friderichsen syndrome due to meningococcemia, trauma, lupus erythematosus, antiphospholipid syndrome, panarteritis nodosa, and anticoagulant therapy. Although microthrombosis and hypercoagulability have been identified in COVID-19, an association with AI is yet to be established.

Secondary AI is caused by tumors of the hypothalamic-pituitary region or by their treatment with surgery or radiotherapy. The most common cause of tertiary AI is the abrupt cessation of high-dose GC therapy owing to the suppression of the hypothalamic-pituitary-adrenal axis by chronic GC administration (11). All individuals receiving a GC dose comparable with ≥20 mg/day of prednisone for more than 2 weeks, or ≥5 mg of prednisone for more than 3-4 weeks, can present suppression of ACTH/CRH release.

The major risk to patients with AI is the absence of normal cortisol responses to stress. Patients with primary AI also demonstrate aldosterone deficiency, which confers an additional risk to the adrenal crisis. All patients with AI should be educated to increase (at least double) the GC replacement dose during stress conditions (infections, trauma, surgery, emotional stress) (11). However, most patients are often extremely reluctant to follow these “sick day rules”. In addition to educational strategies, all patients with AI should maintain an identification card stating the AI diagnosis, as well as the recommendation of receiving 100 mg of hydrocortisone intravenously in case of any medical emergency.

Adrenal crisis is a life-threatening medical condition and remains an important cause of death in patients with AI (12). Even in patients educated regarding appropriate measures in stress conditions, an adrenal crisis is frequent (8.3 cases in 100 individuals with AI per year), with a mortality of 6% (13). Adrenal crisis is defined as an acute deterioration in the health status, associated with hypotension (systolic blood pressure <100 mmHg) that resolves shortly after parenteral GC administration (14). GC replacement therapy for AI consists of the administration of 30 mg/day of hydrocortisone (20 mg early morning and 10 mg at 2-3 pm) or 5-7.5 mg/day of prednisone. Patients with secondary AI might require lower GC doses (20 mg/day of hydrocortisone). Mineralocorticoid replacement (0.05-0.1 mg/day of fludrocortisone) is indicated for patients with primary AI. Patients with AI and symptoms of COVID-19 should immediately double the dose of GC replacement therapy.

During the COVID-19 pandemic, in the presence of any emergency warning signs (such as trouble breathing, persistent pain or pressure in the chest, confusion or cyanosis) or an inability to administer oral GC doses, we recommend that patients with AI should immediately seek an Emergency service to determine the COVID-19 status and receive 100 mg hydrocortisone by intravenous injection, followed by 50 mg hydrocortisone intravenously every 6h or 200 mg/day of hydrocortisone by continuous intravenous infusion. Recently, it has been demonstrated using pharmacokinetic parameters that a continuous intravenous hydrocortisone infusion should be favored over intermittent bolus administration in the prevention and treatment of adrenal crisis during major stress (15). The recommendations of GC therapy for patients with AI and a suspected or confirmed COVID-19 diagnosis are listed in Table 1.

Patients using supraphysiological GC doses are at risk of developing complications of COVID-19 owing to metabolic and cardiovascular complications (hypertension, obesity, and diabetes) associated with chronic GC therapy. If the underlying inflammatory or autoimmune disease is well controlled, the GC dose should be tapered at the earliest. Patients prescribed supraphysiological GC doses presenting any emergency warning signs or an inability to administer the oral medication should receive intravenous hydrocortisone, based on the same recommendation as that for AI.

### GC therapy for critically ill patients with COVID-19

Considering the inflammation and cytokine storm in severe COVID-19, GC treatment for ARDS and septic shock associated with SARS-CoV-2 infection has been debated. To date, there is no published data available on the use of GC in patients with COVID-19 and shock or ARDS. Therefore, a recent recommendation by the European Society of Intensive Care Medicine and the Society of Critical Care Medicine has been based on indirect evidence from critically ill patients in general (16).

A systematic review of 22 randomized controlled trials comparing low-dose GC therapy versus no GC therapy in adults with septic shock failed to demonstrate any significant difference in mortality; however, length of ICU and hospital stays were shortened with GC therapy (17). Moreover, the Surviving Sepsis Campaign COVID-19 panel has suggested using intravenous hydrocortisone (200 mg) per day, administered either as an infusion or intermittent doses, for COVID-19 and refractory shock. In ARDS, evidence of GC use is substantially conflicting because of markedly heterogeneous etiologies and data (16). Finally, in the case of mechanically ventilated adults with COVID-19 and ARDS, several experts have preferred not to issue a recommendation for GC use until higher-quality data are available.

## AUTHOR CONTRIBUTIONS

Ameida MQ was responsible for the manuscript conception and writing. Mendonca BB was responsible for the manuscript conception and critical review.

## Figures and Tables

**Table 1:** Recommendation of GC therapy for adrenal insufficiency (AI) patients with suspected or confirmed COVID-19 diagnosis.

COVID-19 symptoms	Glucocorticoid (GC) dose
**Mild symptoms:**	
Fever, tiredness, dry cough,aches and pains, nasal congestion, runny nose, sore throat, diarrhea.	Double GC dose as soon as symptoms start: - 40 mg hydrocortisone early morning and 20 mg hydrocortisone at 2-3 pm. Consider an additional dose of 20 mg at night if severely fatigued.or - 10 mg prednisone early morning. Consider an additional dose of 2.5-5 mg at night if severely fatigued.- For patients with primary AI: maintain the usual dose of fludrocortisone (0.05-0.1 mg/day on average).
**Warning symptoms:**	
Trouble breathing, persistent pain or pressure in the chest, confusion, or cyanosis.	- Hydrocortisone (100 mg) by intravenous injection in bolus, followed by - Hydrocortisone (50 mg) intravenously every 6h or 200 mg/day by continuous intravenous infusion.

## References

[1] Wu Z, McGoogan JM (2020). Characteristics of and Important Lessons From the Coronavirus Disease 2019 (COVID-19) Outbreak in China: Summary of a Report of 72314 Cases From the Chinese Center for Disease Control and Prevention. JAMA.

[2] Guan WJ, Ni ZY, Hu Y, Liang WH, Ou CQ, He JX (2020). Clinical Characteristics of Coronavirus Disease 2019 in China. N Engl J Med.

[3] Tay MZ, Poh CM, Renia L, MacAry PA, Ng LFP (2020). The trinity of COVID-19: immunity, inflammation and intervention. Nat Rev Immunol.

[4] Whyte CS, Morrow GB, Mitchell JL, Chowdary P, Mutch NJ (2020). Fibrinolytic abnormalities in acute respiratory distress syndrome (ARDS) and versatility of thrombolytic drugs to treat COVID-19. J Thromb Haemost.

[5] Dolhnikoff M, Duarte-Neto AN, de Almeida Monteiro RA, Ferraz da Silva LF, Pierre de Oliveira E, Nascimento Saldiva PH (2020). Pathological Evidence of Pulmonary Thrombotic Phenomena in Severe COVID-19. J Thromb Haemost.

[6] Grillet F, Behr J, Calame P, Aubry S, Delabrousse E (2020). Acute Pulmonary Embolism Associated with COVID-19 Pneumonia Detected by Pulmonary CT Angiography. Radiology.

[7] Zhang Y, Xiao M, Zhang S, Xia P, Cao W, Jiang W (2020). Coagulopathy and Antiphospholipid Antibodies in Patients with Covid-19. N Engl J Med.

[8] Connors JM, Levy JH (2020). COVID-19 and Its Implications for Thrombosis and Anticoagulation. Blood.

[9] Annane D, Bellissant E, Sebille V, Lesieur O, Mathieu B, Raphael JC (1998). Impaired pressor sensitivity to noradrenaline in septic shock patients with and without impaired adrenal function reserve. Br J Clin Pharmacol.

[10] Bancos I, Hahner S, Tomlinson J, Arlt W (2015). Diagnosis and management of adrenal insufficiency. Lancet Diabetes Endocrinol.

[11] Bornstein SR, Allolio B, Arlt W, Barthel A, Don-Wauchope A, Hammer GD (2016). Diagnosis and Treatment of Primary Adrenal Insufficiency: An Endocrine Society Clinical Practice Guideline. J Clin Endocrinol Metab.

[12] Puar TH, Stikkelbroeck NM, Smans LC, Zelissen PM, Hermus AR (2016). Adrenal Crisis: Still a Deadly Event in the 21st Century. Am J Med.

[13] Hahner S, Spinnler C, Fassnacht M, Burger-Stritt S, Lang K, Milovanovic D (2015). High incidence of adrenal crisis in educated patients with chronic adrenal insufficiency: a prospective study. J Clin Endocrinol Metab.

[14] Rushworth RL, Torpy DJ, Falhammar H (2019). Adrenal Crisis. N Engl J Med.

[15] Prete A, Taylor AE, Bancos I, Smith DJ, Foster MA, Kohler S (2020). Prevention of adrenal crisis: cortisol responses to major stress compared to stress dose hydrocortisone delivery. J Clin Endocrinol Metab.

[16] Alhazzani W, Moller MH, Arabi YM, Loeb M, Gong MN, Fan E (2020). Surviving Sepsis Campaign: Guidelines on the Management of Critically Ill Adults With Coronavirus Disease 2019 (COVID-19). Intensive Care Med.

[17] Rygard SL, Butler E, Granholm A, Moller MH, Cohen J, Finfer S (2018). Low-dose corticosteroids for adult patients with septic shock: a systematic review with meta-analysis and trial sequential analysis. Intensive Care Med.

